# Post-Intensive Care Syndrome in Pediatric Neurovascular Patients: A Qualitative Study of Parent Experiences

**DOI:** 10.7759/cureus.100424

**Published:** 2025-12-30

**Authors:** Rya Muller, Sunny Abdelmageed, Ewa Bieber, Sandi Lam, Jonathan Scoville

**Affiliations:** 1 Neurological Surgery, Northwestern University Feinberg School of Medicine, Chicago, USA; 2 Psychiatry, Ann and Robert H. Lurie Children's Hospital of Chicago, Chicago, USA; 3 Neurological Surgery, Ann and Robert H. Lurie Children's Hospital of Chicago, Chicago, USA

**Keywords:** neurocritical care, neurovascular, pediatric, post-intensive care syndrome, qualitative

## Abstract

Background: Post-intensive care syndrome-pediatric (PICS-p) describes long-term physical, cognitive, emotional, and social health impacts seen in pediatric ICU survivors. These symptoms can extend to family members (PICS-f) and have long-term impacts. PICS-p in neurosurgery patients has not been characterized. This pilot project used qualitative methodology to describe PICS in pediatric neurovascular patients and their families.

Materials and methods: Using our single institution's pediatric neurosurgery database, patients who had surgery following an acute neurovascular condition from 2022 to 2023 were identified. Parents/legal guardians of patients were eligible for this qualitative study. Semi-structured interviews explored patients' experiences in the ICU, PICS symptoms among patients and families, and quality of life following discharge from the ICU. Thematic analysis of transcripts was conducted.

Results: Six parents of neurovascular surgery patients were interviewed. The mean age at surgery was 14 (range 11-18) years old. The mean length of ICU stay was 10.8 (range 1-28) days; 50% (N=3) of parents reported symptoms of confusion or diagnosed delirium in their child while in the ICU, 83.3% (N=5) of parents reported PICS symptoms in their child, and 50% (N=3) reported PICS symptoms within their family. The mean follow-up was 17.2 months (range 10.3-25.2). Thematic analysis revealed four themes related to parents' experience in the ICU, the short- and long-term impact of PICS symptoms, and how families adapted to life after hospitalization.

Conclusions: Patients and families continued to experience symptoms of PICS over a year after discharge from the ICU, which has a significant impact on quality of life. Awareness of PICS is essential. Future studies are warranted.

## Introduction

Patients in the ICU who survive critical illness can have significant morbidity [1]. Multiple factors may contribute, including the consequence of the critical illness itself, the consequence of the invasive treatments required to sustain life, and/or the ICU environment, which places a wide range of stressors on patients (e.g., fear, pain, sleep disturbances, sedation, delirium) [2,3]. The post-intensive care syndrome (PICS) framework was developed to conceptualize long-term new or worsening physical, cognitive, and mental health impacts seen in ICU survivors and their families (PICS-family or PICS-f) [4]. Symptoms have been reported in patients across medical and surgical disciplines and include physical weakness, poor sleep, fatigue, difficulties with attention, concentration, and memory, anxiety, depression, and post-traumatic stress symptoms [1]. Impairments frequently persist for months to years following ICU discharge and can have long-lasting impacts [1]. This is especially true for pediatric patients who experience critical illness and hospitalization during a time of significant physical, cognitive, emotional, and social development (PICS-pediatric or PICS-p) and for whom the well-being of the family is directly associated with positive outcomes [5]. It is vital to understand the impact of PICS on both the patient and their family [6].

Brain injuries, invasive treatment, and long hospitalization put neurological patients at an increased risk of developing PICS [7]. A 2018 focus study of pediatric neuro-critical care survivors identified significant symptoms in all PICS domains in patients and their families for years following discharge [8]. This study also highlighted a lack of PICS-related knowledge among medical providers. Hartman et al. cited the important role of pediatric neurologists in the early recognition and management of PICS-p [7]. However, there are no published studies of PICS in neurosurgery patients. This pilot study aims to evaluate the ICU experience and presence of PICS-p and PICS-f in pediatric neurosurgical patients. This study aims to determine whether pediatric neurovascular patients experience PICS, identify areas for future research on this topic, and assess the impact of the ICU and hospitalization on this patient population.

## Materials and methods

Qualitative methodology was used to assess the presence and impact of PICS symptoms in neurovascular patients and their families.

Sample selection, recruitment, and data collection

This retrospective study was approved by the Ann and Robert H. Lurie Children's Hospital Institutional Review Board (approval number: 2023-6351). A convenience sample was used for this study, comprising all patients who underwent surgery for a neurovascular condition at our institution, identified from a neurosurgical procedure database at LCH between 1/1/2022 and 8/23/2023.

Inclusion criteria were (1) being a parent or legal guardian of a child who underwent open cranial surgery for a neurovascular condition (<18 years of age) between 2022 and 2023; (2) being English-speaking without the need for a translator; and (3) having the ability to provide verbal informed consent. Exclusion criteria were (1) the patient did not undergo neurovascular surgery between 2022 and 2023; (2) age ≥18 years; (3) speaking a language other than English; and (4) the patient or parent/legal guardian declined to participate in the study.

Patients who appeared to meet the initial eligibility screening were called by a research assistant (RA) to inform them of the opportunity to participate. A total of N=12 patients were identified during this time period and contacted by telephone to participate. A total of N=6 consented to an interview, which was scheduled and conducted by a second blinded RA over Health Insurance Portability and Accountability Act (HIPAA) compliant teleconferencing. Participants did not receive any monetary compensation for their participation. Patients were given general information about the study, including the goal of learning about the impacts of the surgical journey for neurovascular conditions on patients and families. Following the interview, patient families were provided with information about PICS and offered resources (compiled by author E.B.). The average duration of the interviews was nine minutes and seven seconds. Recordings were automatically transcribed utilizing the transcription feature of Microsoft Teams™ version 4.15.1 (Microsoft Corp., Redmond, WA, USA) and edited as needed for accuracy by the interviewer.

Sociodemographic and baseline information

Clinical (time in days elapsed from surgery) and sociodemographic data (age and race/ethnicity) were collected from the electronic medical record. ICU length of stay was captured during the interview.

Semi-structured interviews

Semi-structured interviews (Appendices) were conducted to understand the parent-reported experience in the ICU and the prevalence of PICS symptoms in both patients and their families. Questions were open-ended; non-directed prompts were used when necessary.​​​​​​​

Analysis

Descriptive statistics were utilized to analyze age at surgery, length of ICU stay, and length of follow-up. A team of two analysts (RM, SA) systematically examined the transcripts through thematic analysis [9]. Inductive coding was performed. These authors individually assigned initial codes using the thematic analysis software ATLAS.ti version 6.0.3 (ATLAS.ti Scientific Software Development GmbH, Berlin, Germany). After completion of initial coding for two interviews, the codes were reviewed, and disagreements were resolved. Codes were maintained in a codebook. Individual coding was then carried out for all remaining interviews. Once all interviews were completed, codes underwent a comprehensive analysis and were organized into themes and subthemes. Inter-coder reliability was assessed using Cohen's kappa, which was 0.269. Cohen's kappa. This is an exploratory pilot with six interviews to inform future studies.

## Results

Patient demographics are shown in Table 1. Six patients were included (two females). The mean age at surgery was 14 (range 11-18) years old. Five were non-Hispanic White patients, and one was a Hispanic patient. By the inclusion criteria, all were primarily English speaking without the need for translators. The mean length of ICU stay was 10.8 (range 1-28) days; 50% (N=3) of parents reported symptoms of confusion or diagnosed delirium in their child while in the ICU, 83.3% (N=5) of parents reported PICS symptoms in their child, and 50% (N=3) reported PICS symptoms within their family. The mean follow-up was 17.2 months (range 10.3-25.2 months).

**Table 1:** Patient demographics NHW: non-Hispanic White, ICU: intensive care unit, PICS: post-intensive care syndrome

Patient	Age (years)	Sex	Race/ethnicity	Length of ICU stay (days)	Follow-up time (months)	Confusion/delirium	Symptoms of PICS
1	12	Male	NHW	1	25.2	No	Yes
2	15	Female	NHW	2	20.9	No	No
3	11	Male	Hispanic	10	16.4	No	Yes
4	13	Female	NHW	5	14.6	Yes	Yes
5	15	Male	NHW	28	15.9	Yes	Yes
6	18	Male	NHW	19	10.3	Yes	Yes

Parent interviews: PICS and the ICU experience

Four themes related to the ICU experience and PICS-p (Table 2) were identified during parent interviews: (1) ICU experience, (2) PICS-p, (3) PICS-f, and (4) life after hospitalization. Parents described challenging experiences in the ICU; however, they were grateful for the treatment they received and their healthcare team. The majority of patients and families reported long-lasting PICS symptoms. Some parents reported difficulty during the recovery period and expressed attempts to move forward with their lives and adapt to their new normal.

**Table 2:** Themes with associated subthemes and participant exemplars PICS: post-intensive care syndrome, PICS-p: post-intensive care syndrome-pediatrics, PICS-f: post-intensive care syndrome family

Theme	Subtheme	Exemplar
ICU experience	Positive experiences with the healthcare team improved the ICU experience	"We had several of (the child’s) neuro team reach out to us via telephone/zooms and a lot of them showed came to visit her while she was (in-patient) and then they had several follow-ups afterwards via zoom and phone calls." (Participant 2) "(The healthcare providers) were really good. They were very supportive and always taking good care of (the child)." (Participant 3) "The nursing staff were very attentive. I remember at one point (the child) tried to get out of her bed because she woke up in the middle of the night and she still had all her tubes. So after that, they gave us a special person to sit with her so we could sleep. We were very well taken care of." (Participant 4) "I would say our experience was good, under the circumstances. Obviously, we don't ever wish this upon any other family, but while we were there, I feel like we had exceptional care, great communication with the PICU doctors and the other consults that were taking care of (the child)." (Participant 6) "I felt very well informed and treated very well." (Participant 1)
	The ICU experience was difficult	"So, you know, (the ICU experience) was stressful because (the cihld) was confused and uncertain (at) the beginning of the whole process. It was a disheartening." (Participant 4) "(The child) was in there for a ruptured brain aneurysm, so I feel like it was just kind of a roller coaster - some days are better than others. I think he was scared, anxious - as we all were." (Participant 6) "The experience of being in the ICU? Scary." (Participant 5) "You know, (the ICU experience was) pretty emotional. Well, I think it was just the seriousness of the whole situation that was very emotional for (the child). I think it was also very painful for him. (When) he was recovering it was emotional because he was not close to his siblings, and he was not able to see them for a while." (Participant 3) "For me and my husband it was painful to see him lying there in the bed and not be able to help him, and also to coordinate time off and trying to work things out (in terms of) taking care of him and the other kids." (Participant 3)
	Support was a positive factor	"We had amazing support with friends, family and, community." (Participant 6) "Well, (our community was) very supportive. We fortunately live close to a lot of families, so they were helpful with my other children and always able to provide assistance if needed." (Participant 2) "I never left (the child’s) side, so there wasn’t really a need for another adult (in the ICU)." (Participant 1)
	Confusion/delirium in the ICU was common	"I remember at one point she tried to get out of her bed because she woke up in the middle of the night and she still had all her brain tubes. So after that, they gave us a special person to sit with her so we could sleep. We were very well taken care of." (Participant 4) "He was out of it the whole time. He wasn't talking. He wasn't walking. Like he was making movements, but they weren’t like super like meaningful movements. Umm, like he was awake, but he wasn't there..." (Participant 5) "Yes. He definitely had some delirium - that was hard, just trying to reorient him, keep him in bed, get him what he wanted to try to calm him down. And that lasted at least a few days." (Participant 6)
	Neurological deficits were challenging	"When she figured out she couldn't see - she lost part of her vision - she was scared. And then when she first woke up the first time, she was more scared of what was happening to her body." (Participant 4) "I mean, he's he still has seizures, but he has no deficits." (Participant 4) "(The impact of treatment is) less and less every month you get out there. But it's affected our lives - the vision thing obviously doesn't go away and (the child) gets older and closer to driving, and now she can't play contact sports." (Participant 4) "We're just in a really rough patch right now because he was doing so great post-op when we left (the hospital). But as of two weeks ago he was in the emergency room in college, and we just found out that he's having seizures. So, he had to withdraw from school and so now we're just in a full medical workup, which we started last week to try to figure out how keep the seizures under control. So at the moment it's been affecting us with work, he can't go to school right now and we're trying to figure out what his path will be for working and everyday life and activity.” (Participant 6)
PICS-p	Cognitive symptoms were most common	"He has had challenges with reading challenges with pretty much anything that applies to reading, being able to handle and understand different questions that are asked of him from the reading, characters, plot narratives, point of view, stuff like that. So anything with English altogether, English language art has been a challenge." (Participant 1) "Concentrating is hard. It takes a little while for him to learn something comfortably, like before we would tell him a problem and he would get the answer right away and now it takes more time for him to get it. And he doesn’t really focus as well. He does get frustrated with this because then he is thinking of his limitations, he says thinks like oh because I have this, I can’t do things like I used to back then." (Participant 3)
	Emotional symptoms	"Maybe emotionally like he was always very like happy and playful and now like I don’t know he has become a little bit more sad. Especially when he starts talking about it." (Participant 3) "She's more self-aware about, you know, she calls herself the blind kid. And she says, you know, “I almost died,” - we'll talk about that once in a while - so she certainly aware and that comes up at least a few times a week, most of those, if not more." (Participant 4) "I would say (the impact of treatment is) day-to-day depending on how he’s feeling. So, we could have like a great day or a day where you're just like at home in the dark, lights off, you know. Just every day is different." (Participant 5)
	Social symptoms	"Yes, I'd say there has been less (social) interactions after his hospitalization." (Participant 5) "She kind of, you know, took a few months to get back, but she's back to exactly where she was socially and academically." (Participant 4)
	Physical symptoms were less common	"So after yeah after surgery, he has gotten like a few headaches where, like, he just he doesn't really get up from the bed from like a day or two, you just pretty much laid on the bed. He gets nauseous sometimes and so he doesn’t really have as much of an appetite. (He) Definitely (has) worse coordination. I don’t know (why but) he has become very clumsy though." (Participant 3)
PICS-f	Emotional symptoms were the most common	"I would say that now with him or I guess with all of my kids I am more worried or anxious about their health. For example, if my kids bring up having some sort of pain it is just more serious. My little one had a headache similar to what my son had and that freaked me out a lot, oh my god, I kept being really on her and trying to get it checked out and searching online because I was worried it might be the same situation. I guess I have always been a little like that but now I think it has increased my worry when any of them have a health issue." (Participant 3) "I always wonder, you know, because this isn't something that really has any symptoms or warning that was caught just by chance through an MRI. So, it's always something in the back of your mind. Like, could they have it? You won't know unless you know they ever need a scan and find it." (Participant 2)
	Social symptoms	"When he got out of the hospital it was hard because people were starting to find out and there were a lot of people (to handle) at once. And people started saying I can’t believe this, and saying (things) like oh it was nothing and it was not a big deal. So it was really uncomfortable to bear their comments because a lot of them didn’t know exactly what he went through." (Participant 3)
	Cognitive symptoms	"I want to say (my child was in the ICU) four or five days. I can't remember exactly. It could have been longer." (Participant 4)
	Physical symptoms were less common	"You know, it was exhausting. It was, you know, it took a lot out of both of us, physically and mentally. The doctors, nurses and staff were great. I mean, it's just the stress of what your child's going through. You know, three or four weeks after we (were admitted to the) ICU, when I got home, I was sick for a couple of months with flu that I couldn't shake, I think, because my body was so beaten up. So, I think it took a toll physically." (Participant 4)
	Emotional symptoms were reported in siblings	"Yes actually. Her older sister was having some mental health issues and part of it is anxiety about health issues about myself - I've had a few open-heart surgeries - and (the patient), because of what she went through, I think is partly why. So, she's seeing somebody for that right now. But I would say is you know to some degree certainly related to what happened to (the patient)." (Participant 4) "Well, you know, with his siblings then, they were a little smaller; they didn’t know much. In the first days they were okay but towards the end they were getting a little bit scared since he was away from them and actually, we were all away from them since we were with my child." (Participant 3)
Life after hospitalization	Grateful for treatment	"As I said before, (the diagnosis) comes up and we talk about it and say, 'I can't believe that happened' and 'how lucky you are' and you know 'what if we didn't get into the hospital.'" Participant 4 "We're very fortunate. I keep saying that, but we really, really are. I know so many other families that go to (hospital) and we are very fortunate that we've had such a great outcome." (Participant 2) "This is our life, and this is just how we move forward. I would say we move forward with positivity and gratitude. So, we just are kind of just getting used to our new normal." (Participant 1)
	Moving forward and adjusting to their new normal	"We talk about it as something that happened, that we're moving forward, and this is just like our new normal. This is our life, and this is just how we move forward, and I would say we move forward with positivity and gratitude. We are just getting used to our new normal." (Participant 1) "We just take it one year at a time on a follow up and just keep moving forward as and I hesitate to say normal but as a normal life." (Participant 2)
	Open dialogue	"We discuss (the hospitalization within our family) openly. Yeah. I mean, as I said before, it comes up and we talk about it and say, 'I can't believe that happened' and 'how lucky you are' and you know 'what if we didn't get into the hospital' - that kind of stuff. So always an open dialogue." (Participant 4) "(When discussing the hospitalization within our family) I guess we just sort of give forth information and feel free to answer questions if anyone has questions." (Participant 2)
	Desired support was variable after discharge	"I think for him (support or resources we desire) would be therapy as far as different techniques to actually use what is remaining of his vision and coping with it, because now he has been dealing with it pretty much on his own. I think he would benefit a lot from it. But no one reached out, I feel like they weren’t able to provide me referrals and the referrals they did give to me weren’t accurate or some of them didn’t even exist anymore." (Participant 3) "(The support has) tapered off. I'm trying to find someone to help her more with her vision, and I wasn't getting very far. I've emailed back and forth with the neurologist, and it's not as attentive as before. We're going to have a test in a month or two, but you know, I wish there was a little more checking in although maybe there doesn't need to be. However, after going through something like that it's hard to just drop it." (Participant 4) "No, (I do not desire additional support or resources). I feel like we had a lot of support after we left the hospital through, like (rehab hospital) and therapies and so forth. And still continued (now)." (Participant 5) "No, (I do not desire additional support or resources). I still feel like at any time if I do have a question, I can reach out to her neuro nurse and she's always quick to answer any questions I have." (Participant 2)

Theme 1: ICU Experience

Although parents found the ICU experience difficult, they were grateful to have access to care and described various factors that influenced their ICU experience.

The ICU experience was challenging: Parents describe the ICU experience as “scary,” “emotional,” “painful,” and “stressful” with one parent reporting, “for me and my husband it was painful to see him lying there in the bed and not be able to help him.” Parents also reported that the ICU experience was challenging for their child, with one mother stating, “(the ICU experience was) pretty emotional. I think it was just the seriousness of the whole situation that was very emotional for (the child). I think it was also very painful for him. (When) he was recovering, it was emotional because he was not close to his siblings and wasn’t able to see them for a while.”

Confusion/delirium in the ICU was common: Parents reported symptoms of confusion or diagnosed delirium while their child was in the ICU. One parent stated, “He was out of it the whole time. He wasn't talking. He wasn't walking. He was making movements, but they weren’t super meaningful movements. He was awake, but he wasn't there.” Another parent expressed difficulty dealing with their child’s delirium: “He definitely had some delirium - that was hard, just trying to reorient him, keep him in bed, get him what he wanted to try to calm him down. And that lasted at least a few days.”

Neurological deficits were challenging: Postoperative neurological deficits impacted parents and their children, with one parent explaining, “When she figured out she couldn't see - she lost part of her vision - she was scared. And then when she first woke up the first time, she was more scared of what was happening to her body.”

Positive experiences with the healthcare team improved the ICU experience: Parents report feeling “very well taken care of.” Although the ICU experience was challenging, one parent reports, “our experience was good, under the circumstances. Obviously, we don't ever wish this upon any other family, but while we were there, I feel like we had exceptional care, great communication with the PICU doctors and the other consults that were taking care of (the child).”

Support was a positive factor: Support from various systems was helpful to parents during their child’s ICU stay. As one mother expressed, “Well, (our community was) very supportive. We fortunately live close to a lot of families, so they were helpful with my other children and always able to provide assistance if needed.”

Theme 2: PICS-p

Parents reported PICS symptoms experienced by the patient (Table 3). Cognitive symptoms were most commonly reported (66.7%, n=4), followed by emotional (50%, n=3), social (33.3%, n=2), and physical (16.7%, n=1) symptoms.

**Table 3:** PICS symptoms seen in patient PICS: post-intensive care syndrome

	Cognitive	Emotional	Social	Physical
1	Yes	No	No	No
2	No	No	No	No
3	Yes	Yes	No	Yes
4	No	Yes	Yes	No
5	Yes	Yes	Yes	No
6	Yes	No	No	No

Cognitive symptoms were the most common: Parents reported cognitive changes in their child following their ICU experience. Parents noted difficulty with school, noting, “(the child) has had challenges with pretty much anything that applies to reading, being able to handle and understand different questions that are asked of him from the reading, characters, plot narratives, point of view.” Parents also described difficulty with learning and attention with one mother stating, “concentrating is hard. It takes a little while for him to learn something comfortably. Before we would tell him a problem and he would get the answer right away and now it takes more time for him to get it. And he doesn’t really focus as well. He does get frustrated with this because then he is thinking of his limitations, he says things like ‘oh because I have this, I can’t do things like I used to back then.’” 

Emotional symptoms: Parents reported noticing emotional differences in their child following their ICU experience. One parent expressed, “he was always very happy and playful and now he has become a little bit more sad. Especially when he starts talking about (the hospitalization).” Parents also reported symptoms of fixation in their child, stating, “She's more self-aware - she calls herself the blind kid. And she says, ‘I almost died,’ - we'll talk about that once in a while - so she certainly aware and that comes up at least a few times a week, most of those, if not more.”

Social symptoms: Parents reported “less social interactions” after their child's hospitalizations. Some parents reported that these symptoms initially occurred and improved over time, stating, “She took a few months to get back, but she's back to exactly where she was socially and academically.”

Physical symptoms were the least commonly reported: Physical challenges reported included lack of energy and changes in appetite. One parent stated, “After surgery, he has gotten like a few headaches where he doesn't really get up from the bed for a day or two. He gets nauseous sometimes and so he doesn’t really have as much of an appetite. (He) definitely (has) worse coordination. I don’t know (why but) he has become very clumsy.”

Theme 3: PICS-f

PICS symptoms in the family of the patient were also reported (Table 4). Emotional symptoms were most commonly reported (50.0%, N=3), followed by cognitive (33.3%, N=2), social (16.7%, N=1), and physical (16.7%, N=1). In the parents, PICS symptoms were reported in all domains. In siblings, only emotional symptoms were reported.

**Table 4:** PICS symptoms seen in family PICS: post-intensive care syndrome

	Cognitive	Emotional	Social	Physical
1	No	No	No	No
2	Yes	Yes	No	No
3	No	Yes	Yes	No
4	Yes	Yes	No	Yes
5	No	No	No	No
6	No	No	No	No

Emotional symptoms were the most common: Parents reported feeling increased anxiety about their child and their siblings, with one parent stating, “I would say that now with him, or I guess with all of my kids, I am more worried or anxious about their health. For example, if my kids bring up some sort of pain it is just more serious. My little one had a headache similar to what my son had and that freaked me out a lot. I kept being on her, trying to get it checked out and searching online because I was worried it might be the same situation. I guess I have always been a little like that but now I think it has increased my worry when any of them have a health issue.”

Cognitive symptoms: Parents also reported difficulty with memory. One parent reported, “I want to say (my child was in the ICU) four or five days. I can't remember exactly. It could have been longer.”

Social symptoms: Parents describe feeling isolated from their community, stating, “When (the child) got out of the hospital it was hard because people were starting to find out and there were a lot of people to handle at once. And people started saying ‘I can’t believe this,’ and ‘oh it was nothing’ and ‘it was not a big deal.’ So, it was really uncomfortable to bear their comments because a lot of them didn’t know exactly what he went through.”

Physical symptoms were the least common: following the ICU experience, patients reported feeling “exhausted.” One parent stated, “It was exhausting. It took a lot out of both of us, physically and mentally. The doctors, nurses and staff were great. It's just the stress of what your child's going through. Three or four weeks after we (were admitted to the) ICU, when I got home, I was sick for a couple of months with flu that I couldn't shake because my body was so beaten up. So, I think it took a toll physically.”

Emotional symptoms were reported in siblings: Parents reported their child's siblings feeling “scared” during the ICU experience. Parents also reported increased anxiety in their child’s siblings following the ICU experience, with one parent explaining, “Her older sister was having some mental health issues and part of it is anxiety about health issues about myself - I've had a few open-heart surgeries - and (the patient), because of what she went through. So, she's seeing somebody for that right now. But I would say it is to some degree certainly related to what happened to (the patient).”

Theme 4: Life Following Hospitalization

The ICU experience and hospitalization significantly impacted families. However, when reflecting on life afterward, most parents described attempting to come to terms with what happened and adjust to their new reality.

Parents were grateful for the treatment: Reflecting on their ICU experience, parents were grateful for the healthcare provided. One mother expressed, “We're very fortunate. I keep saying that, but we really, really are. I know so many other families that go to (hospital) and we are very fortunate that we've had such a great outcome.” Additionally, parents expressed feeling fortunate for life-saving care, with one parent stating, “(when the diagnosis) comes up and we talk about it and say, ‘I can't believe that happened’ and ‘how lucky you are’ and ‘what if we didn't get into the hospital.’”

Parents described moving forward and adjusting to their new normal: Parents expressed trying to “move forward,” stating, “We talk about it as something that happened, that we're moving forward, and this is just like our new normal. This is our life, and this is just how we move forward, and I would say we move forward with positivity and gratitude. We are just getting used to our new normal.”

Open dialogue: Following the hospitalization, parents attempted to have an open dialogue surrounding the ICU experience and diagnosis with their child and family. One parent expressed, “We give forth information and feel free to answer questions if anyone has any.”

Desired support varied after discharge: Some parents expressed a desire for increased support and healthcare coordination. One parent stated, “(The support has) tapered off. I'm trying to find someone to help (the child) more with her vision, and I wasn't getting very far. I've emailed back and forth with the neurologist, and (they’re) not as attentive as before. We're going to have a test in a month or two, but you know, I wish there was a little more checking in although maybe there doesn't need to be. However, after going through something like that it's hard to just drop it.” Other parents reported that they were doing well and did not desire additional support or resources, stating, “I feel like we had a lot of support after we left the hospital through (the rehab facility) therapies and so forth. And still continued (now).” Parents also commented on the availability of the healthcare team, with one parent stating, “I still feel like at any time if I do have a question, I can reach out to her neuro(surgery) nurse and she's always quick to answer any questions I have.”

## Discussion

PICS describes long-term new or worsening cognitive, social, emotional, and physical symptoms in ICU survivors [4]. Up to 80% of patients in the pediatric ICU (PICU) experience decreased health-related quality of life (HRQoL) following discharge [10,11]. The convergence of brain injury and invasive interventions increases the risk of PICS in neurological patients [12]. Over 50% of pediatric patients with neurologic injuries experience physical disabilities, neurocognitive deficits, and psychosocial challenges within the first year following discharge [13,14]. No research has been published on the prevalence of PICS in neurosurgical populations, despite the majority of neurosurgical patients spending time in the ICU. This pilot study demonstrates that neurosurgical patients experience PICS symptoms, underscoring the importance of provider recognition, prevention, and treatment.

Our study identified symptoms of PICS-p and PICS-f at 10.3-25.2 months following ICU discharge, which is consistent with the chronicity of PICS described in the literature. Within our cohort, emotional symptoms, followed by cognitive impairments, were most commonly reported by both patients and family members. Similar to previous research on cognitive symptoms following hospitalization [15], our patients most frequently experienced difficulties with focus and processing information. Parents endorsed emotional symptoms, including depression and anxiety. However, it is unclear if these are stand-alone symptoms or if they are a part of a PTSD syndrome, as we did not specifically screen for this. Symptoms of PTSD, particularly depression and sleep disturbances, are highly prevalent (83% and 80%, respectively) among PICS survivors [16,17]. Fifty percent of our study’s parents reported emotional disturbances, consistent with findings from larger studies [18,19]. This is particularly relevant as parental mental health has been identified as an essential factor in the parents' ability to support their child’s recovery [20]. Parental distress has been associated with a higher risk of child distress [21].

Delirium is a neuropsychiatric disorder characterized by acute disturbances in alertness and attention and at least one other cognitive function [2,3]. Fifty percent of parents surveyed in our study reported their child had been diagnosed with delirium or experienced episodes of confusion while in the ICU. Delirium is associated with increased morbidity, duration of mechanical ventilation, and longer hospital stays [22], which may increase the risk of PICS. In published studies, no correlation has been drawn between the length of stay and the number of PICS symptoms experienced [7]. Future studies are warranted to investigate the effect of delirium on the prevalence of PICS-p and PICS-f symptoms.

We found that 33.3% and 16.7% of the parents interviewed in our study reported their child experiencing social and physical PICS symptoms, respectively. It has been reported that PICU patients have lower overall social and physical HRQoL scores than healthy matched controls following discharge [23]. Additionally, older age at admission and longer length of stay are associated with worse physical and social HRQoL at 12-month follow-ups [24]. Social impacts also extend to the family, as restricted activities and financial stress impact the family’s abilities to engage in social activities [8]. The development of PICS-p support groups has been previously suggested as a method of treatment for psychosocial symptoms; further research is needed [8].

Recognition, management, and, if possible, prevention of PICS are vital to prevent long-term symptoms. One proposed strategy is the ICU bundle, also known as the ABCDEF bundle (assess, prevent, and manage pain; both spontaneous awakening and breathing trials; choice of analgesia and sedation; preventing and managing delirium; early mobility and exercise; family engagement and empowerment) [25]. The ABCDEF bundle is a harm-reduction tool that was developed to manage symptoms in adult ICU patients, reducing the incidence of delirium, ICU readmission, duration of mechanical ventilation, and duration of coma [25]. Modified pediatric ABCDEF protocols have been proposed, with limited data on their implementation or efficacy [26]. Across 161 PICUs in the United States, Canada, Brazil, and Europe, only nine had incorporated all six parts of the ABCDEF bundle [27]. Further research is needed on the ABCDEF bundle and the prevention of PICS in pediatric patients and families.

A population of neurosurgery patients receives care in the intensive care unit and are at-risk patients for PICS. Awareness of PICS is essential. Neurosurgeons can ensure that PICS symptoms are addressed through multidisciplinary collaboration with psychiatrists and neurologists. Furthermore, neurosurgeons follow their patients longitudinally and are positioned to recognize PICS symptoms as they arise. By preventing PICS or addressing PICS symptoms early, neurosurgeons can maximize patients' quality of life and reduce morbidity both in the ICU setting and beyond.

For patients continuing to experience neurological deficits and PICS symptoms, parents expressed a desire for more healthcare coordination. Studies have demonstrated that parents struggle with the limited availability of PICS resources [8]. Parents have expressed desires for increased provider knowledge on PICS, improved communication between specialists and primary care providers following discharge, and more educational materials [8]. In this study, most parents were not aware of PICS prior to their interview and appreciated the PICS resources provided.

Future directions

This pilot study with parents demonstrates that PICS occurs in pediatric patients undergoing neurosurgical treatment of neurovascular disease. No comments can be made on its prevalence. The association between neurological condition, neurosurgical intervention, length of ICU hospitalization, presence of delirium, and the impact of sociodemographic factors and PICS warrants evaluation to understand risk factors for PICS among neurosurgical patients.

Currently, there are no validated screening tools for PICS-p. Previous studies have used a combination of screening tools, none of which cover all four components of PICS-p independently [28]. Additionally, only two tools are available to assess PICS-f [28-30]. A validated screening tool for PICS is vital to identifying PICS in clinical settings by various stakeholders, including neurosurgeons. Standardization of PICS diagnosis is needed to obtain accurate prevalence estimates and to guide further research.

Limitations

There are limitations to this study. This study is limited by its small sample size and may be subject to selection bias. Roughly one-third of patients in our neurosurgery practice are Spanish-speaking, so this study captures only English-speaking families interested in participating in this pilot. Additionally, these interviews provided retrospective parental perspectives and, thereby, might be subject to recall bias and may not fully reflect the child’s subjective experience. Future research should evaluate PICS across a larger population of neurosurgical patients and a variety of pathologies. Open-ended questions were used rather than a directed screening tool, which may have led to some PICS symptoms not being captured.

Nevertheless, this is the first study to describe PICS and the journey of neurovascular patients and their families through the ICU. By utilizing a qualitative framework, this study is an initial step to opening the discourse on PICS in pediatric neurosurgery and identifies future directions for further study. Recognition of the PICS-p framework is a start.

## Conclusions

PICS symptoms were identified in this pilot study of pediatric neurovascular patients treated by neurosurgery. Patients and families continued to experience symptoms of PICS over a year after discharge from the ICU. Neurosurgeons care for at-risk patients and their families. Awareness of PICS is essential. Future studies should investigate risk factors for PICS among neurosurgical patients and their families and explore strategies for prevention and treatment.

## Appendices

**Table 5:** Questions used during semi-structured interview regarding PICS, experiences in the pediatric ICU, and the long-term impact of this experience on patients and families PICS: post-intensive care syndrome, ICU: intensive care unit

Topic	
PICS	PICU: How long was your child in the intensive care unit? What was your child’s experience in the intensive care unit? What was your family’s experience there? Did your child experience any confusion or disorientation in the ICU? Were there any times they seemed frightened by their environment? Did they ever experience hearing or seeing things others did not? Were you provided any information about these symptoms? How was your child and your family supported through this experience? How has the neurovascular treatment affected any experiences in your day-to-day lives? Were there any unexpected ways in which hospitalization impacted your life? Post-PICU: After leaving the hospital, what, if any, changes have you experienced with your child? What physical symptoms? Have you noticed any changes in their emotions or behavior? How about changes to their attention or thinking? What about changes in their social interactions? Have there been any such changes in family members, including yourself? How does your family talk about the diagnosis, surgery, and hospitalization? Since leaving the hospital, have there been any additional supports or resources you wished were offered to your child or family members?
